# Splenic macrophage functional profile and its role in the immunopathogenesis of canine visceral leishmaniasis

**DOI:** 10.3389/fimmu.2025.1617751

**Published:** 2025-06-20

**Authors:** Tainã Luís de Souza, Marta de Almeida Santiago, Francini Neves Ribeiro, Fabiano Borges Figueiredo, Artur Augusto Velho Mendes Junior, Rodrigo Caldas Menezes, Renato Porrozzi, Fernanda Nazaré Morgado

**Affiliations:** ^1^ Laboratório de Imunoparasitologia, Instituto Oswaldo Cruz, Fundação Oswaldo Cruz, Rio de Janeiro, Brazil; ^2^ Laboratório de Protozoologia, Instituto Oswaldo Cruz, Fundação Oswaldo Cruz, Rio de Janeiro, Brazil; ^3^ Laboratório de Biologia Celular, Instituto Carlos Chagas, Fundação Oswaldo Cruz, Curitiba, Brazil; ^4^ Laboratório de Pesquisa Clínica em Dermatozoonoses em Animais Domésticos, Instituto Nacional de Infectologia Evandro Chagas, Fundação Oswaldo Cruz, Rio de Janeiro, Brazil

**Keywords:** visceral leishmaniasis, dog, cell exhaustion, macrophage, spleen, M1 macrophages, M2 macrophages

## Abstract

**Introduction:**

Visceral leishmaniasis (VL) represents a major public health challenge, with the spleen frequently identified as one of the primary target organs. Dogs recognized as urban reservoir hosts, commonly harbor chronic infections characterized by elevated parasite burdens across multiple tissues. This study aims to analyze functional markers of M1 and M2 responses, as well as PD-L1+ macrophages in the spleens of naturally infected dogs with Leishmania infantum, and to correlate these findings with splenic white pulp disorganization, parasitic load, and clinical severity.

**Methods:**

Thirty-four VL-infected dogs were enrolled, each undergoing clinical evaluation to determine a clinical severity score. Histopathological analyses were performed to evaluate splenic white pulp disorganization, while quantitative PCR and immunohistochemistry were employed to assess parasite burden. Immunological markers were analyzed via immunohistochemistry, immunofluorescence, and flow cytometry.

**Results:**

Splenic white pulp disorganization was observed in most animals, indicating marked tissue disruption. Immunostaining demonstrated the presence of NOS2^+^, Arginase 1^+^, pSTAT3^+^, CD206^+^, and TGF-β^+^ cells, reflecting the engagement of both M1 and M2 macrophage subsets in the immune response, with a predominance of M1 profile. Elevated CD206 expression correlated with splenic white pulp disruption and parasite load. A notable finding was the decrease in the CD68^+^NOS2^+^/CD68^+^Arginase-1^+^ ratio in animals with higher parasite load. Additionally, significant PD-L1 expression was detected in macrophages within spleens exhibiting splenic white pulp disorganization, indicative of a pro-exhaustion cellular phenotype. Flow cytometry analysis identified co-expression of arginase-1 and PD-L1, as well as Arginase-1^high^+ cells. Finally, arginase-1high+ cells directly correlated with arginase-CD14-PD-L1^+^ cells suggesting that not only macrophages, but others arginase-1high expressing cell types may contribute for suppressive/regulatory profile during the immunopathogenesis of canine VL.

**Conclusion:**

The persistent presence of CD206, CD68^+^Arginase-1^+^ and CD68^+^PD-L1^+^ cells within the inflamed, parasitized splenic tissue, alongside a relative decline in CD68^+^NOS2^+^ cells, may create a permissive environment for parasite survival and replication, thereby sustaining the inflammatory response. This chronic exposure to antigenic and inflammatory stimuli likely contributes to persistent tissue damage, exemplified by splenic white pulp disorganization in the spleen, and exacerbates disease progression.

## Introduction

Visceral Leishmaniasis (VL) is a major public health problem due to the large number of people at risk of infection and the high lethality when left untreated (1). In Brazil, it is caused by the protozoan *Leishmania infantum* (2), and human and animal cases are expanding geographically (3, 4). In urban environments, domestic dogs are considered major reservoirs because they can harbor a high parasite load even in healthy skin (5), and the detection of canine cases often precedes the emergence of human cases (6, 7).

Loss of parasite control correlates with a failure of the immune response in dogs (8). In Brazil, the treatment of infected dogs with meglumine antimoniate, the drug of choice for Human Visceral Leishmaniasis (HVL), is prohibited (9). However, in other countries, treatment is allowed (10, 11) but is often associated with a high relapse rate (10, 12). The transition from infection to active Canine Visceral Leishmaniasis (CVL) is marked by a prominent humoral response, suppression of cellular immunity, and the onset of clinical signs (13). Disease progression has been associated with splenic architectural disorganization (11, 14, 15), increased parasite load, and decreased cytokine and chemokine expression (16). It was found in our previous studies that animals with high parasite loads exhibit not only follicular atrophy but also marked reductions in both pro- and anti-inflammatory cytokine mRNA expression (17), a profile consistent with cellular exhaustion.

T cell exhaustion, initially observed in chronic viral infections, is characterized by increased expression of the inhibitory receptor PD-1 (Programmed Death-1), often detectable early in infection (18). PD-1 expression is triggered by repeated antigenic stimulation in T and B lymphocytes, while its ligand, PD-L1, is constitutively expressed by B cells, T cells, macrophages, and dendritic cells in the spleen (19). PD-1 activation promotes apoptosis, inhibits proliferation, and suppresses cytokine production (20). In our previous research, we identified PD-1, TIM-3, LAG-3, and CTLA-4 expression in the spleens of infected dogs (21), and noted increased apoptotic CTLA-4^+^ cells in disorganized spleens correlating with high parasite burden (21).

The substantial parasite loads in advanced stages of infection (8), along with diminished lymphoproliferative responses, may involve immune suppression mechanisms such as IL-10 and TGF-β activity. Macrophages, the main host cells for *Leishmania*, may contribute to CVL immunopathogenesis. Disruption of the splenic white pulp may compromise not only CD4 T cells but also macrophage functionality.

Macrophages are the cells where *Leishmania* reproduces and survives for long periods (22–24). A protective immune response against these parasites is predominantly introduced by Th1 cells, which are key to activating the macrophage oxidative stress machinery, resulting in the production of reactive oxygen and nitrogen species and disease control (25). NOS2 and arginase-1 share the same substrate (L-arginine), competing for its availability, and arginase inhibition in *L. donovani*-infected human macrophages enhances NOS2 expression and vice versa (26). While NOS2 is an important marker for M1 macrophage activation, Arginase-1 expression indicates an M2 phenotype. A recent review (27) explains that a balance between M1 and M2 macrophages is crucial: excessive M1 activation causes tissue damage, whereas a high M2 profile promotes parasite growth. CD206, the mannose receptor, is also used as a M2 profile marker (28). Macrophages and dendritic cells can express it as a surface receptor or in its soluble form (29). In acute liver failure, it can be considered a biomarker of severity (29). STAT3 is one of the transcription activator factors involved in the polarization of M2 macrophages (28). In visceral leishmaniasis, the expression of IFN-γ, Arginase-1 and STAT3 impaired macrophage and T lymphocytes responses *in vitro* (30).

M2 macrophages producing IL-10 and TGF-β have been identified in the spleens of dogs with VL (31), and PD-L1+ macrophages have been observed in the spleens of *L. donovani*-infected mice (32). Therefore, this study aims to analyze functional markers of M1 and M2 responses, as well as PD-L1+ macrophages, in the spleens of dogs naturally infected with *Leishmania infantum* and correlate these findings with splenic white pulp disorganization (SWPD), parasite load, and clinical scores.

## Materials and methods

### Ethics statement

Dogs naturally infected with *L. infantum*, tested seropositive for anti-*Leishmania* antibodies by the rapid dual-path platform assay (TR DPP, Biomanguinhos, Fiocruz, Rio de Janeiro, Brazil) and by enzyme immunoassay (ELISA, Biomanguinhos, Fiocruz, Rio de Janeiro, Brazil) were included in the study and scheduled for euthanasia between 2013 and 2014 according to the guidelines of the Brazilian Ministry of Health. All dog owners provided formal written consent for their participation. Sample collection occurred during necropsies performed by veterinarians at the Laboratório de Pesquisa Clínica em Dermatozoonoses em Animais Domésticos-INI-Fiocruz (LAPCLIN-DERMZOO-INI/Fiocruz). This study received approval from the Comitê de Ética em Uso de Animais (CEUA-Fiocruz) under license LW-54/13 and adhered to Brazilian Law 11794/08 and the guidelines of the Sociedade Brasileira de Ciência em Animais de Laboratório (SBCAL). The access to genetic heritage was registered under number AFB4BD9 on the SisGen platform.

### Animals

Thirty-four dogs from Barra Mansa, Rio de Janeiro, Brazil, diagnosed with *L. infantum* infection and referred for mandatory euthanasia at the Evandro Chagas National Institute of Infectious Diseases (INI/FIOCRUZ) were included. Euthanasia was carried out by veterinarians according to the following protocol: a) Intravenous administration of 1.0% thiopental (Tiopentax^®^, Cristalia) at a dosage of 1.0mL/kg; and b) After confirming the absence of a corneal reflex induced by deep anesthesia, 10mL of 19.1% potassium chloride (Isofarma) was administered intravenously. Spleen and blood samples were collected during necropsy; clinical data were recorded beforehand by two veterinarians.

### Clinical evaluation

Clinical signs of CVL were evaluated per established protocols (21), assessing dermatitis, onychogryphosis, ophthalmic changes, body condition loss, alopecia, and lymph adenomegaly. Each sign was graded from 0 (absent) to 3 (severe) (33), and the total clinical score was used to classify animals as having low (0–2), moderate (3–6), or high (7–18) clinical severity.

### Histopathological analysis

Spleen tissue fragments were formalin-fixed in a 10% formalin-buffered solution (Merck, Darmstadt, Germany) and embedded in paraffin (Synth, Diadema, Brazil). Histological sections (5 μm thick) were mounted on microscopic slides and stained with hematoxylin and eosin (Leica Biosystems, Newcastle Upon Tyne, UK) for subsequent analysis using an optical microscope (Zeiss, Oberkochen, Germany). The organization of the splenic lymphoid tissue, including the white pulp, marginal zone, and red pulp, was assessed as previously described (14). The white pulp was graded as follows: 1) Organized, characterized by a periarteriolar sheath, germinal centers, and distinct mantle and marginal zones. 2) Slightly disorganized, exhibiting some hyperplastic or hypoplastic changes resulting in a partial loss of definition of certain white pulp regions. 3) Moderately disorganized, with white pulp regions barely individualized or indistinct. 4) Intensely disorganized, where the follicular structure is scarcely distinguishable from the red pulp and the T cell area. Then, the quantification of lymphoid follicles per square millimeter of tissue was conducted. This evaluation considered both the presence or absence of follicles and the number of lymphoid follicles per field.

### Extraction of DNA from splenic tissue

Total DNA was extracted from approximately 10 mg of spleen using the QIAmp DNA Mini Kit (Qiagen, Santa Clarita, USA), so involving an initial digestion step with proteinase K (20 mg/mL) for 1 hour at 56°C. The DNA was then eluted in TE buffer and quantified using a NanoDrop^®^ spectrophotometer (Thermo Fisher Scientific, Waltham, USA).

### Determination of the parasite load by qPCR

Parasite load in the spleen was quantified using real-time PCR following established protocols (17). HPRT primers (see **Supplementary Table S1**) were used to normalize the canine DNA content in each sample. Quantification of *Leishmania* DNA was performed using primers targeting the small subunit ribosomal RNA (ssrRNA) gene, a multi-copy gene commonly used for parasite detection (34) (see **Supplementary Table S1**).

qPCR assays were conducted using a Step One instrument (Applied Biosystems, Molecular Probes, Inc., Foster City, USA) with Power SYBR Green Master Mix (Applied Biosystems, Molecular Probes, Inc., Foster City, USA). Two microliters of purified total DNA (100 ng) were added to a final PCR volume of 20 μl, containing Power SYBR Green 1X and 300 nM of each primer for HPRT PCR assays or 500 nM of each primer for ssrRNA PCR assays. The qPCR protocol involved an activation step at 95°C for 10 minutes, followed by 40 cycles of denaturation and annealing/extension (95°C for 15 seconds, 60°C for 1 minute, and 68°C for 30 seconds).

A standard curve was generated for each target. All reactions were performed in duplicate for each target, and both targets were run on the same plate for the same sample. For quantification, peripheral blood mononuclear cells (PBMCs) from uninfected dogs and bulk cultures of *L. infantum* promastigotes (strain MCAN/BR/2007/CG-1) were used. PBMCs and promastigotes were quantified using a cell counter (Z1™ COULTER COUNTER^®^, Beckman Coulter, Fullerton, USA), and total DNA was extracted from 1x10^6^ PBMCs and 1x10^7^ promastigotes. Standard curves for the HPRT and ssrRNA genes were prepared using 10-fold serial dilutions. The animals were then categorized into high or low-parasite load groups based on previously established criteria (17).

### Immunohistochemistry

Spleen tissue fragments were embedded in Tissue-Tek OCT resin (Sakura, Alphen aan den Rijn, The Netherlands) and sectioned at a thickness of 5 μm. Sections were mounted on silanized slides (Dako, Carpinteria, CA, USA) using acetone P.A. (Merck), then hydrated in phosphate-buffered saline (PBS) for 10 minutes. Endogenous peroxidase activity was blocked with 3% hydrogen peroxide (Dako) for 1 minute at room temperature. After two 5-minute washes in PBS, non-specific binding was blocked with 0.4% bovine serum albumin (BSA) for 20 minutes at room temperature.

Excess blocking solution was removed, and primary antibodies targeting CD68 (macrophages), CD206 (mannose receptor), NOS2 (nitric oxide synthase 2), TGF-β (transforming growth factor beta), and *Leishmania* amastigotes were applied. Sections were incubated with primary antibodies for 18 hours at 4°C. Control sections were prepared by omitting the primary antibody.

After incubation, sections were washed twice in PBS (5 minutes each) and incubated with the appropriate biotinylated secondary antibody (Dako) for 25 minutes, followed by two more PBS washes. Streptavidin-peroxidase (Genetex) was then applied for 25 minutes. After additional PBS washes, the immunoreactivity was visualized using an AEC substrate kit (Invitrogen, Carlsbad, USA). The reaction was stopped with type II water upon microscopic visualization. Slides were counterstained with Meyer’s hematoxylin (Sigma-Aldrich, Saint Louis, USA) and mounted in Faramount medium (Dako).

Quantification of NOS2, CD206, and TGF-β positive cells was performed in five fields per spleen section at 40× magnification. Amastigote quantification was performed in 20 fields at 1000× magnification. Results are expressed as the median percentage and range (minimum to maximum) of positive cells or positive cells per square millimeter. Qualitative analyses were performed in both the red and white pulp regions.

### Immunofluorescence

Immunofluorescence was conducted as previously described (21). Briefly, splenic tissue sections were fixed in acetone P.A. (Merck) and blocked to reduce non-specific antibody binding. The sections were then incubated with primary antibodies targeting macrophages (Bio-Rad, USA), pSTAT-3, arginase-1 (Cell Signaling Technology, USA), NOS2, PD-L1, CD68 (Abcam, USA), and *L. infantum* antigen. Secondary anti-mouse IgG antibodies conjugated with phycoerythrin (PE-red) and fluorescein isothiocyanate (FITC) were used, along with anti-mouse IgG conjugated with Dylight 633 and anti-mouse IgG conjugated with Dylight 488 or Alexa fluor 488. Slides were mounted with Fluoromount-G medium containing DAPI (4′, 6-diamino-2-phenylindole) (Thermo Fisher Scientific) and examined using a fluorescence microscope. Image processing and overlay analyses were performed using ImageJ software (NIH, USA). The brightness and contrast were adjusted.

### Flow cytometry

Splenocytes were isolated from 1 cm splenic fragments through mechanical disruption and successive washings, then cryopreserved in a solution of 90% fetal bovine serum (FBS) and 10% dimethyl sulfoxide (DMSO; Sigma) and stored in liquid nitrogen until further analysis. Thawing was performed at 37°C, followed by the gradual addition of RPMI medium supplemented with 20% FBS (1 mL to 4 mL increments) at one-minute intervals. Cells were subsequently centrifuged at 1800 rpm for 10 minutes at 7°C to remove the supernatant and resuspended in 5 mL of RPMI with 20% FBS. Following homogenization, an additional 5 mL of RPMI with 20% FBS was added. The samples were centrifugated for 10 minutes at 1800 rpm at 7°C. Cells were incubated on ice for 20 minutes in a blocking solution containing 10% goat serum and 10% FBS to block Fc receptors. After centrifugation, cell viability, and concentration were assessed using a Neubauer chamber and Trypan blue exclusion method and adjusted to 1×10^7^ cells/mL. Only samples exhibiting a viability greater than 60% proceeded to the labeling protocol.

Cell viability was assessed using the Zombie Aqua dye (Invitrogen) for flow cytometry. A total of 1×10^6^ cells per tube were washed with 200 µL PBS and incubated with 100 µL of Zombie dye for 20 minutes at room temperature. After washing with PBS, surface staining was conducted. The primary anti-PD-L1 antibody (Biolegend, USA) was incubated for 20 minutes at 4°C. After washing with PBS/BSA (PBS containing 0.1% BSA, 0.05% sodium azide), the secondary anti-mouse PE antibody (Invitrogen) was added and incubated for 20 minutes at 4°C. After another wash with PBS/BSA, the anti-CD14-APC antibody (BD Biosciences, USA) was added and incubated for 20 minutes at 4°C. Cells were then fixed and permeabilized using Cytofix/Cytoperm solution (BD), followed by washing with a permeabilization buffer (PBS/BSA containing 0.5% saponin). Intracellular staining was performed by resuspending cells in 30 µL of the permeabilization buffer and incubating with anti-arginase-1 PE-Cyanine 7 antibody (Invitrogen) for 30 minutes at 4°C. After a final wash, samples were washed and resuspended in PBS for acquisition in the flow cytometer.

Flow cytometric analysis was performed on a 12-color CytoFLEX flow cytometer equipped with 405 nm, 488 nm, 565 nm, and 638 nm lasers (Beckman Coulter, Brea, CA, USA) at the Multi-user Research Facility of Flow Cytometry – Multiparametric Analysis, Instituto Oswaldo Cruz, FIOCRUZ. Data acquisition was managed using CytExpert software (Beckman Coulter). Fluorochromes were analyzed as follows: PE and PE-Cy7 (excited by 565 nm laser) with filters 585/42 and 780/60, respectively; APC (excited by 638 nm laser) with filter 660/10; and Zombie Aqua (excited by 405 nm laser) with filter 525/40.

Data analysis was conducted using FlowJo software version 7.6.5 (Becton Dickinson, Franklin Lakes, NJ, USA). Initial gating was based on forward scatter (FSC) versus side scatter (SSC) to delineate the cell population, followed by the exclusion of doublets via FSC-A *vs*. FSC-H gating (**Supplementary Figure 1**). Viability was determined using a histogram of Zombie Aqua staining, and only samples greater than 50% viable cells were included. From the live cell population (Zombie-negative, live gate), expression levels of CD14, arginase-1, and PD-L1 were quantified. Single, dual, and triple expression profiles were assessed using the combination gating tool in FlowJo. The combination gating tool is an automatically combination of gates defined in simple analyses which permits all possible combinations (positive/negative), creating ‘AND’, ‘OR’ and ‘NOT’ populations for each antibody, resulting in different cell phenotypes’.

### Statistical analysis

A non-probabilistic sampling approach was employed in this study, with animals classified into three groups based on clinical presentation: (1) low clinical score, (2) moderate clinical score, and (3) high clinical score. Furthermore, animals were stratified according to the degree of organization in splenic lymphoid tissue: (1) organized (ranging from well-organized to mildly disorganized splenic white pulp) and (2) disorganized (ranging from moderately to severely disrupted splenic white pulp). Additionally, animals were categorized based on parasite burden: (1) low and (2) high.

The Shapiro–Wilk test was applied to assess the normality of data distribution. For nonparametric data, comparisons between independent groups were conducted using the Mann–Whitney U test or Kruskal–Wallis test followed by Dunn’s *post hoc* test. For parametric data, analyses were performed using the unpaired Student’s t-test or one-way ANOVA followed by Tukey’s multiple comparisons test. Correlations were assessed using either Pearson’s or Spearman’s rank correlation coefficient, as appropriate. Statistical analyses were conducted using GraphPad Prism version 8.0 (GraphPad Software, San Diego, CA, USA). A p-value less than 0.05 was considered statistically significant. Results are expressed as median and range (minimum–maximum).

## Results

### Clinical score, parasite load and splenic white pulp disorganization in dogs naturally infected with *L. infantum*

Thirty-four dogs naturally infected with *L. infantum* were enrolled in this study. The groups were homogeneously distributed according to age and sex, as presented in the **Table 1**.

**Table 1:** The distribution of sex and age across the study groups.

Clinical Score				
Clinical Data	Low	Medium		High
Sex (number)				
Male	6	8		6
Female	6	7		7
Age (Years)*	3.23 ± 2.82	5.17 ± 3.54		4.23 ± 2.52
Parasite load				
Clinical Data	Low parasite load		High parasiteload	
Sex (number)				
Male	10		10	
Female	10		10	
Age (Years)**	4.25 ± 3.29		4.16 ± 2.72	
Splenic White Pulp Organization				
Clinical Data	Organized to slight disorganization		Moderate to intense disorganization	
Sex				
Male	7		13	
Female	8		13	
Age(Years)***	3.13 ± 1.81		4.94 ± 3.44	

*p = 0.29 (oneway anova); **p= 0.07 (T-test); ***p = 0.93 (T-test).

The most frequently observed clinical manifestations of Canine Visceral Leishmaniasis (CVL) – including dermatitis, alopecia, onycogryphosis, keratoconjunctivitis, lymphadenomegaly, and poor body condition – were evaluated.

Based on the severity of these clinical signs, the animals were categorized into three groups: low (n = 10), medium (n = 13), and high clinical score (n = 11), as previously described (21). Additionally, splenic parasite burden was quantified, and animals were categorized into low (n = 18) and high (n = 16) parasite load groups.

**Figure 1:**
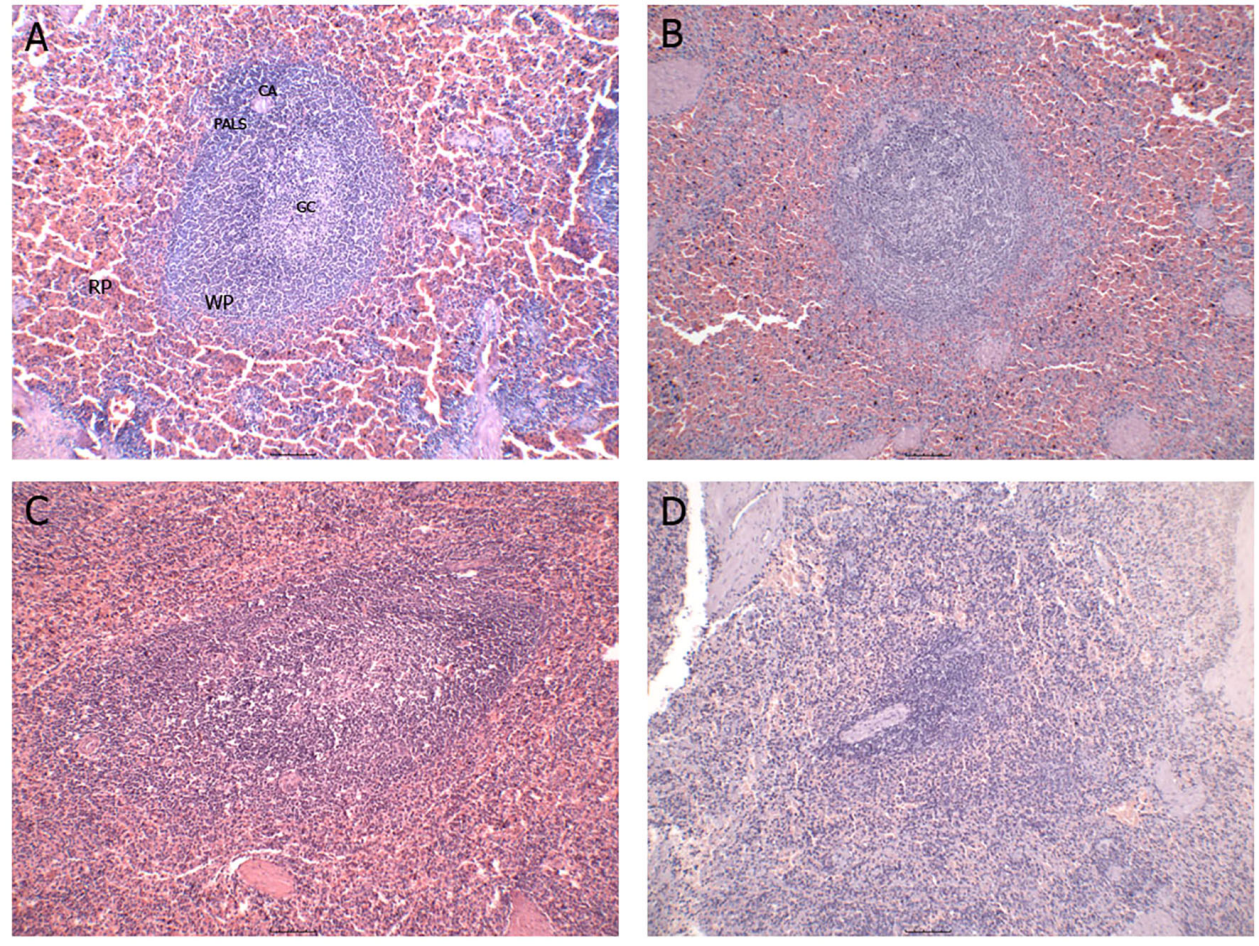
Representative images of the different degrees of splenic white pulp organization/disorganization. **(A)** organized splenic white pulp; **(B)** slightly disorganized splenic white pulp; **(C)** moderately disorganized splenic white pulp; **(D)** severely disorganized splenic white pulp. RP, red pulp; WP, white pulp; CA, central arteriole; PALS, periarteriolar lymphatic sheath; GC, germinal center.

Disorganization of the splenic white pulp was assessed and defined by reducing the number and compartmentalization of lymphoid follicles and the periarteriolar lymphoid sheath (**Figure 1**). A significant proportion of the animals (30/34; 75%) exhibited varying degrees of white pulp disorganization, ranging from mild (n = 9), moderate (n = 10), to severe (n = 11). This histopathological alteration appears to be a common feature and potentially associated with the progression of CVL (21).

### Increased presence of CD206^+^ cells in the spleen of dogs naturally infected with *L. infantum* in relation to splenic white pulp disorganization

**Figure 2:**
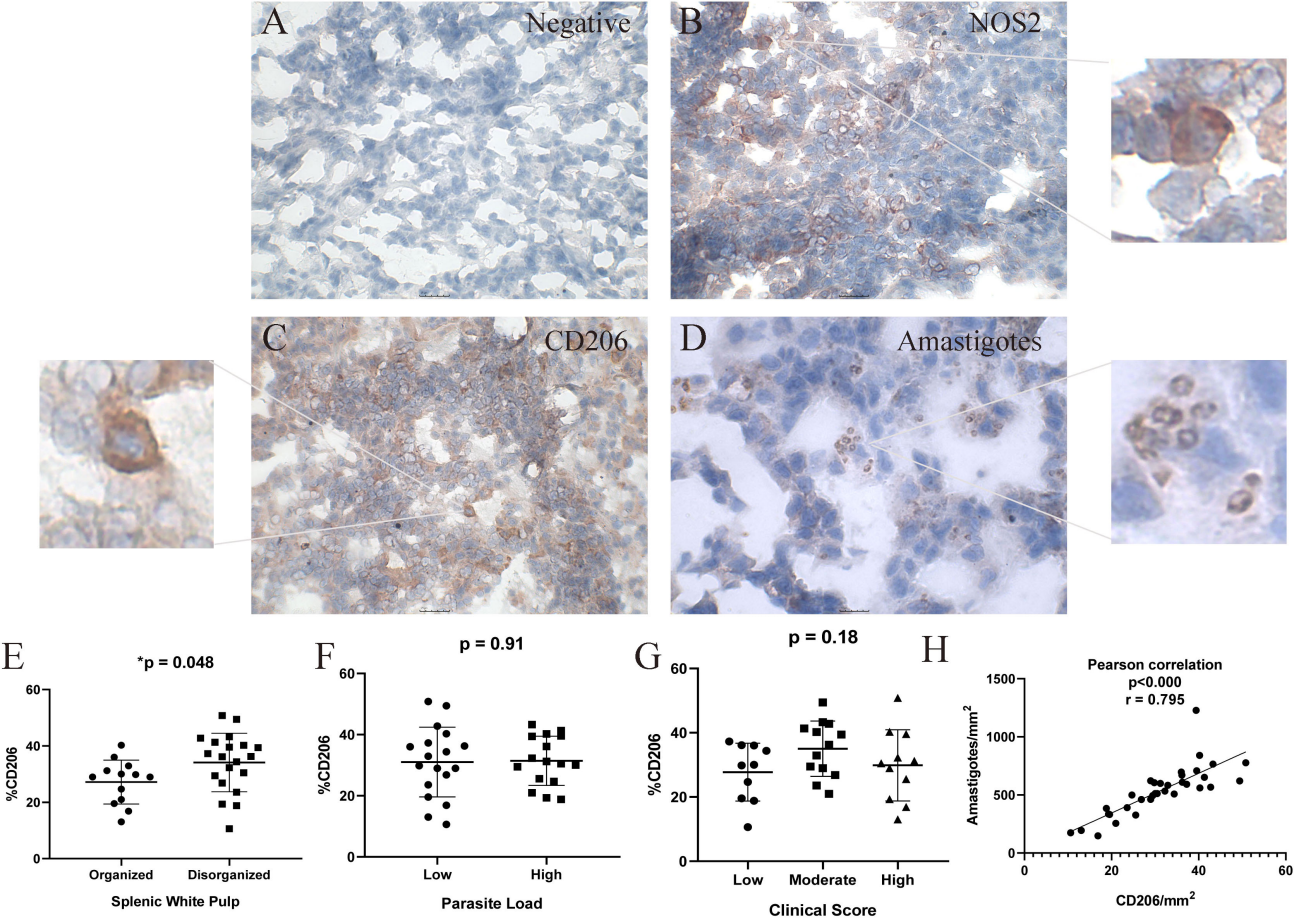
Immunohistochemical Analysis of Spleen Tissue from Dogs Naturally Infected with *Leishmania infantum* for the Detection of Nitric Oxide Synthase 2 (NOS2), CD206, and *L. infantum* Amastigotes. **(A)** negative control, **(B)** nitric oxide synthase 2 (NOS2) and zoom, **(C)** CD206 (mannose receptor) and zoom, **(D)** amastigotes and antigens of L.infantum and zoom in the spleen of naturally infected dogs. Positive cells stained in red. Magnification bar: **(A-C)** 25µm; **(D)** 10 µm. **(E)** percentage of CD206^+^ cells according to splenic white pulp disorganization; **(F)** percent of CD206+ cells according to parasite load; **(G)** percent of CD206^+^ cells according to clinical score; **(H)** Positive correlation between CD206^+^cells/mm2 and amastigotes/mm2. **(E, F)** Unpaired t-test. **(G)** One-way ANOVA. **(H)** Pearson correlation. Quantification of NOS2 and CD206 positive cells was performed in five fields per spleen section at 40× magnification. A total of 500 cells were counted as positive or negative cells. The percentage was calculated as (positive cells x 100) ÷ total counted cells.

Immunohistochemical analysis was performed on splenic sections to detect and quantify the expression of NOS2, TGF-β, and CD206 (mannose receptor), along with the presence of *Leishmania* amastigotes (**Figure 2**, **Supplementary Table S2**). Positive cells for NOS2, CD206, and TGF-β were observed in all animals, indicating the involvement of distinct macrophage profiles in the response to *L. infantum* infection. Overall, NOS2^+^ cells were the most prevalent, compared to CD206^+^ and TGF-β^+^ markers (**Supplementary Table S2**).

We observed two types of CD206 labeling: cellular labeling predominantly in macrophages, but also diffuse labeling in the extracellular medium. Notably, a significantly greater number of CD206^+^ cells was detected in spleens with moderate to severe white pulp disorganization compared to those with preserved or slightly altered architecture (p = 0.048; unpaired t-test) (**Figure 2E**). No statistically significant differences were found in CD206^+^ cell counts when stratified by clinical score or parasite load. However, there was a correlation between CD206/mm^2^ and amastigotes/mm^2^ (p < 0.000; r = 0.795) (**Figure 2H**).

**Figure 3:**
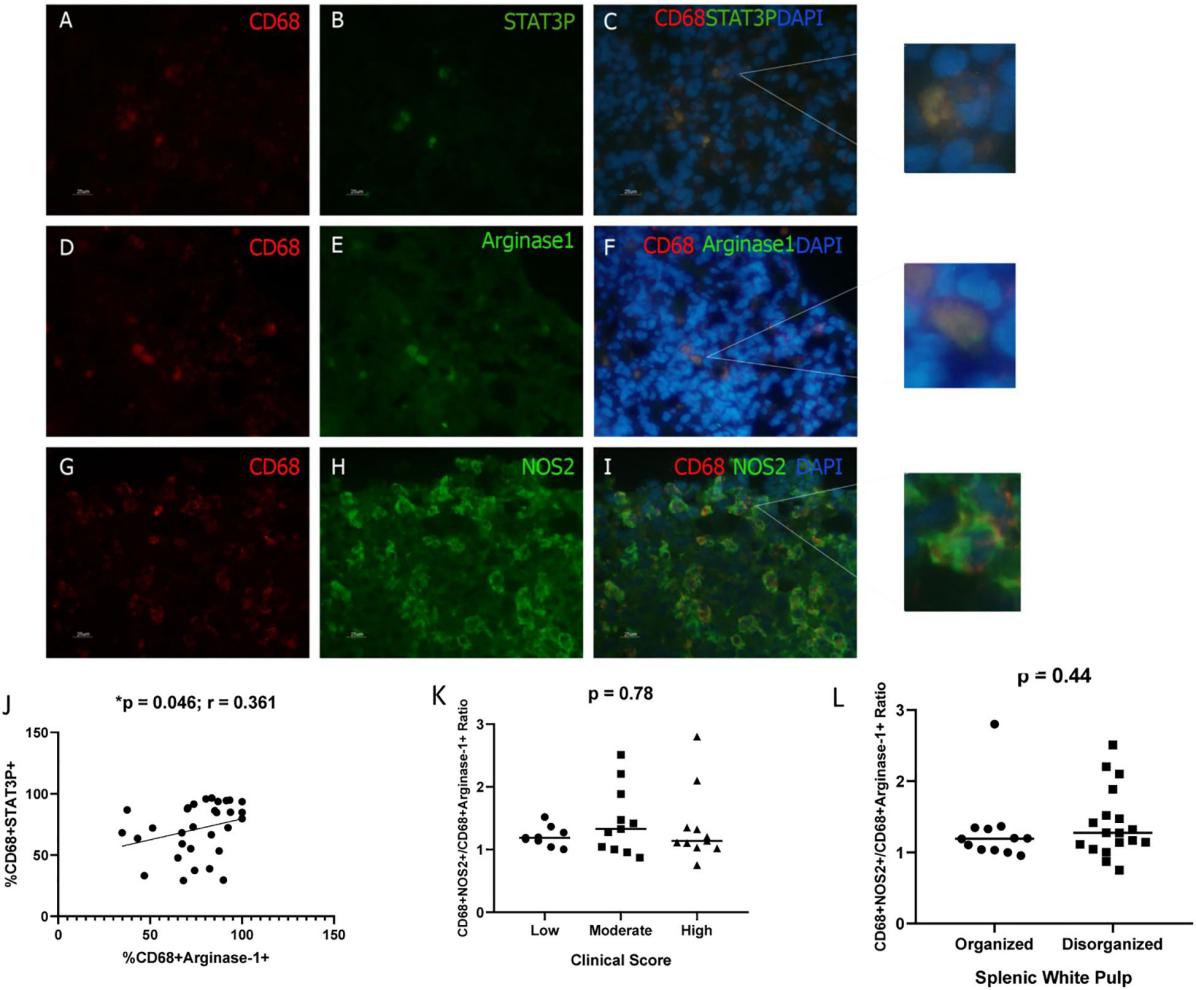
Detection of Macrophages expressing pSTAT3, Arginase-1, or NOS2 in the spleen of dogs naturally infected with *L. infantum*. **(A, D, G)** CD68^+^ cells (Macrophages/PE); **(B)** pSTAT-3 (FITC); **(E)** arginase-1 (FITC); **(H)** NOS2 (FITC); **(C, F, I)** overlapping images and zoom (PE/FITC/DAPI). Magnification bar: 25µm. **(J)** Correlation between CD68^+^STAT3^+^ and CD68^+^arginase-1^+^ cells (Spearman correlation). **(K)** CD68^+^NOS2^+^/CD68^+^arginase-1+ cells ratio according to clinical score (Kruskal-Wallis test); **(L)** CD68^+^NOS2^+^/CD68^+^arginase-1+ cells ratio according to splenic white pulp disorganization (Mann-Whitney test). **(K, L)** The ratio was obtained by dividing the %CD68+NOS2+ cells by %CD68+arginase+ cells for each animal.

Macrophage polarization was further assessed via immunofluorescence. During this analysis, macrophages (CD68^+^ cells) co-expressing phosphorylated STAT-3, arginase-1, or NOS2 were identified (**Figure 3**). No significant differences were detected in the frequencies of CD68^+^pSTAT-3^+^, CD68^+^arginase-1^+^, or CD68^+^NOS2^+^ cells across groups categorized by splenic white pulp organization, clinical score, or parasite burden (**Supplementary Table S2**). However, a positive correlation was observed between CD68^+^pSTAT-3^+^ and CD68^+^arginase-1^+^ cell frequencies (p = 0.046; r = 0.361; Spearman’s correlation).

### Reduced CD68^+^NOS2^+^/CD68^+^arginase-1^+^ cell ratio in the spleens of dogs with elevated parasite load

**Figure 4:**
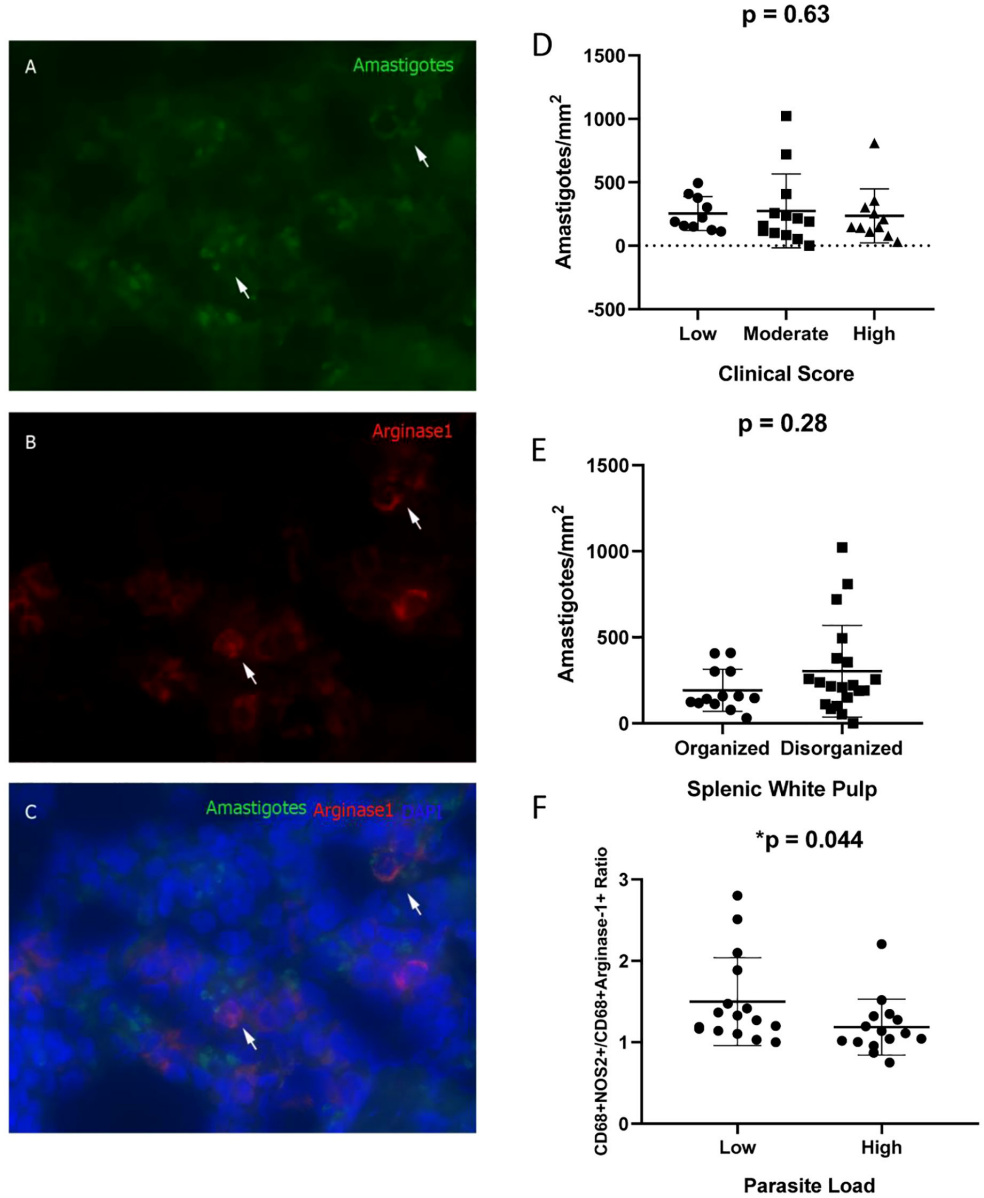
Co-localization of Arginase-1^+^ Cells and *Leishmania infantum* Amastigotes in the Spleens of Naturally Infected Dogs. **(A)** Amastigotes (Alexa Fluor 488); **(B)** Arginase-1^+^ cells (DyLight 633); **(C)** Merged image (Alexa Fluor 488/DyLight 633/DAPI). **(D)** Amastigote density (amastigotes/mm²) stratified by clinical score (Kruskal–Wallis test); **(E)** Amastigote density according to splenic white pulp disorganization (Mann–Whitney test); **(F)** CD68^+^NOS2^+^/CD68^+^Arginase-1^+^ cell ratio based on parasite load (Mann–Whitney test). The ratio was obtained by dividing the %CD68+NOS2+ cells by %CD68+arginase+ cells for each animal.

By using immunofluorescence analysis of spleen sections at 100× magnification, the co-localization of arginase-1^+^ macrophages and *Leishmania infantum* amastigotes was confirmed (**Figure 4**). This observation indicates that arginase-1^+^ macrophages are phagocytic and capable of harboring intracellular amastigotes (**Figure 4C**). Notably, amastigotes were also detected in arginase-1^−^ cells.

No statistically significant differences in amastigote density (amastigotes/mm²) were observed across groups stratified by clinical score or splenic white pulp organization (**Figures 4D, E**). However, animals exhibiting a high parasite load demonstrated a significantly reduced CD68^+^NOS2^+^/CD68^+^Arginase-1^+^ cell ratio (**Table 2**, **Figure 4F**).

**Table 2:** Ratio of NOS2^+^ Macrophages to Arginase-1^+^ Macrophages according to splenic white pulp organization, parasite load, and clinical score in dogs naturally infected with *Leishmania infantum*.

Clinical Score					
Ratio	Low	Medium		High	p-value (Kruskal-Wallis)
NOS2/arginase-1 ratio in macrophages/%**	1.19 (1.0 – 1.52)	1.33 (0.87 – 2.51)		1.14 (0.75 – 2.8)	0.78
Parasite Load					
Ratio	Low parasite load		High parasite load		p-value (Mann-Whitney)
NOS2/arginase-1 ratio in macrophages/%**	1.3 (1.0 – 2.8)		1.11 (0.75 – 2.21)		**0.045**
Splenic White Pulp Organization					
	Organized to slight disorganization		Moderate to intense disorganization		p-value (Mann-Whitney))
NOS2/arginase-1 ratio in macrophages/%**	1.19 (0.96 – 2.8)		1.27 (0.75 – 2.51)		0.44

Data are represented as median (minimum-maximum values).

** Data obtained using immunofluorescence.P-value < 0.05 is represented in bold.

### CD68^+^PD-L1^+^ cells were detected in the spleen of dogs naturally infected with *L. infantum*

**Figure 5:**
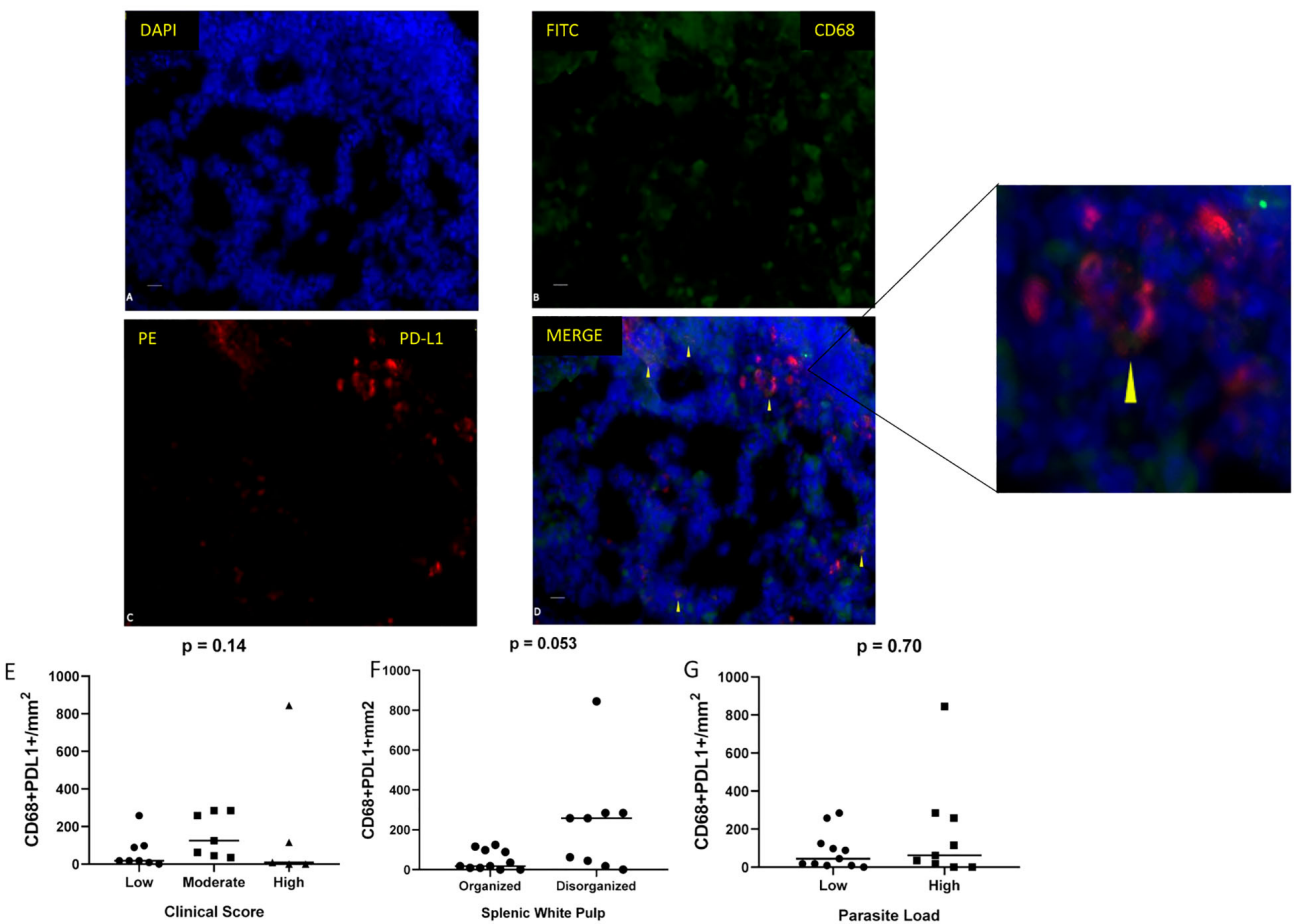
Immunofluorescence detection of macrophages expressing PD-L1 in spleen sections of dogs naturally infected with *L. infantum*. **(A)** DAPI, **(B)** CD68 (FITC), **(C)** PD-L1 (PE), and **(D)** merged images (FITC/PE/DAPI). Scale bar: 25 µm. Quantification of CD68^+^/PD-L1^+^ cells per mm² according to **(E)** clinical score (Kruskal–Wallis test), **(F)** splenic white pulp disorganization (Mann–Whitney test), and **(G)** parasite load (Mann–Whitney test).

Immunofluorescence analysis of spleen sections at 40× magnification revealed *in situ* expression of the exhaustion marker PD-L1 on macrophages (CD68^+^) (**Figure 5**). A trend towards elevated PD-L1 expression was observed in animals exhibiting disorganized splenic white pulp (p = 0.053; Mann–Whitney test) (**Figure 5F**).

### Monocyte infiltration in the spleen is reduced with disease progression

**Figure 6:**
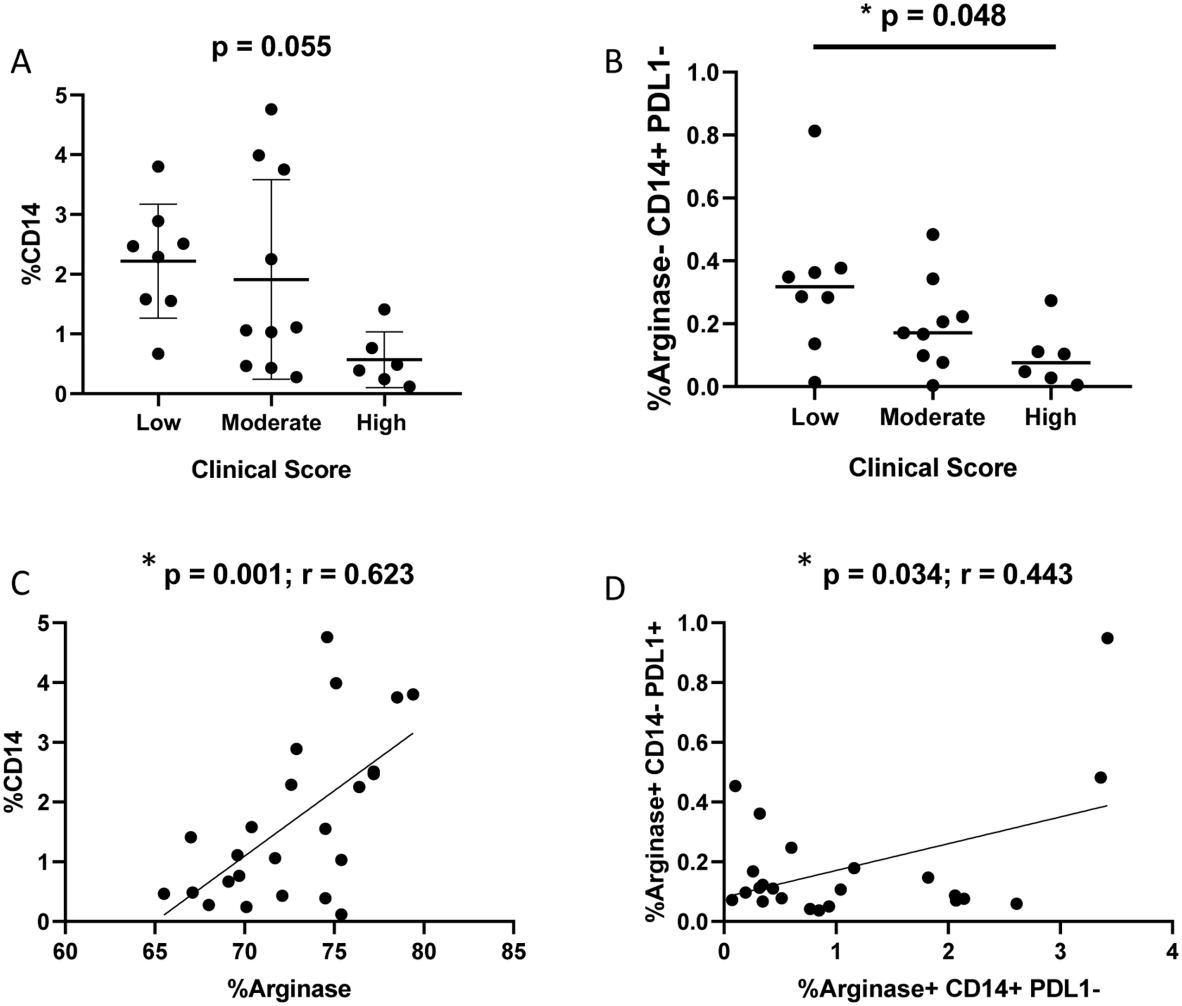
Flow cytometry analysis of splenic cells to detect CD14^+^, PD-L1^+^ and arginase-1^+^ cells according to clinical score. **(A)** Percentage of CD14^+^ cells according to clinical score (Kruskal-Wallis test). **(B)** Percentage of CD14^+^PD-L1^-^Arginase-1^-^ according to clinical score (Kruskal-Wallis test). **(C)** Correlation between %CD14^+^ and %arginase-1^+^ cells (Spearman correlation). **(D)** Correlation between CD14^+^arginase-1^+^PD-L1^-^ and CD14^-^arginase-1^+^PD-L1^+^ cells.

To assess monocyte recruitment in the spleen, CD14^+^ cells were quantified via flow cytometry, alongside the co-expression of arginase-1 and PD-L1 (**Supplementary Table S3**, **Figure 6**). A decrease in the frequency of CD14^+^ cells (p = 0.055; one-way ANOVA) and CD14^+^arginase-1^−^PD-L1^−^ cells (p = 0.048; Kruskal–Wallis test) was observed in dogs with a high clinical score (**Figures 6A, B**). Furthermore, significant correlations were found between CD14^+^ and arginase-1^+^ cells (p = 0.001; r = 0.623; Spearman correlation), as well as between CD14^+^arginase-1^+^PD-L1^−^ and CD14^−^arginase-1^+^PD-L1^+^ cell populations (p = 0.034; r = 0.443; Spearman correlation) (**Figures 6C, D**). In Panel C, the correlation occurs between CD14^+^ cells and total cells expressing arginase-1. Note that the percentage values (X-axis of Panel C) are much higher than those in Panel D because it includes not only M2 cells but also various leukocytes expressing the enzyme, including lymphocytes and neutrophils. In Panel D, the correlation occurs between CD14^+^Arginase-1^+^PD-L1^-^ cells and CD14^-^Arginase-1^+^PD-L1^+^ cells. Notice that the percentages are much lower, as it represents only a fraction of the population demonstrated in Panel C.

### Flow cytometry analysis reveals the presence of arginase-1^high^ cells in the spleen of infected dogs and correlations between arginase-1^high^ and PD-L1 exhaustion pathway

**Figure 7:**
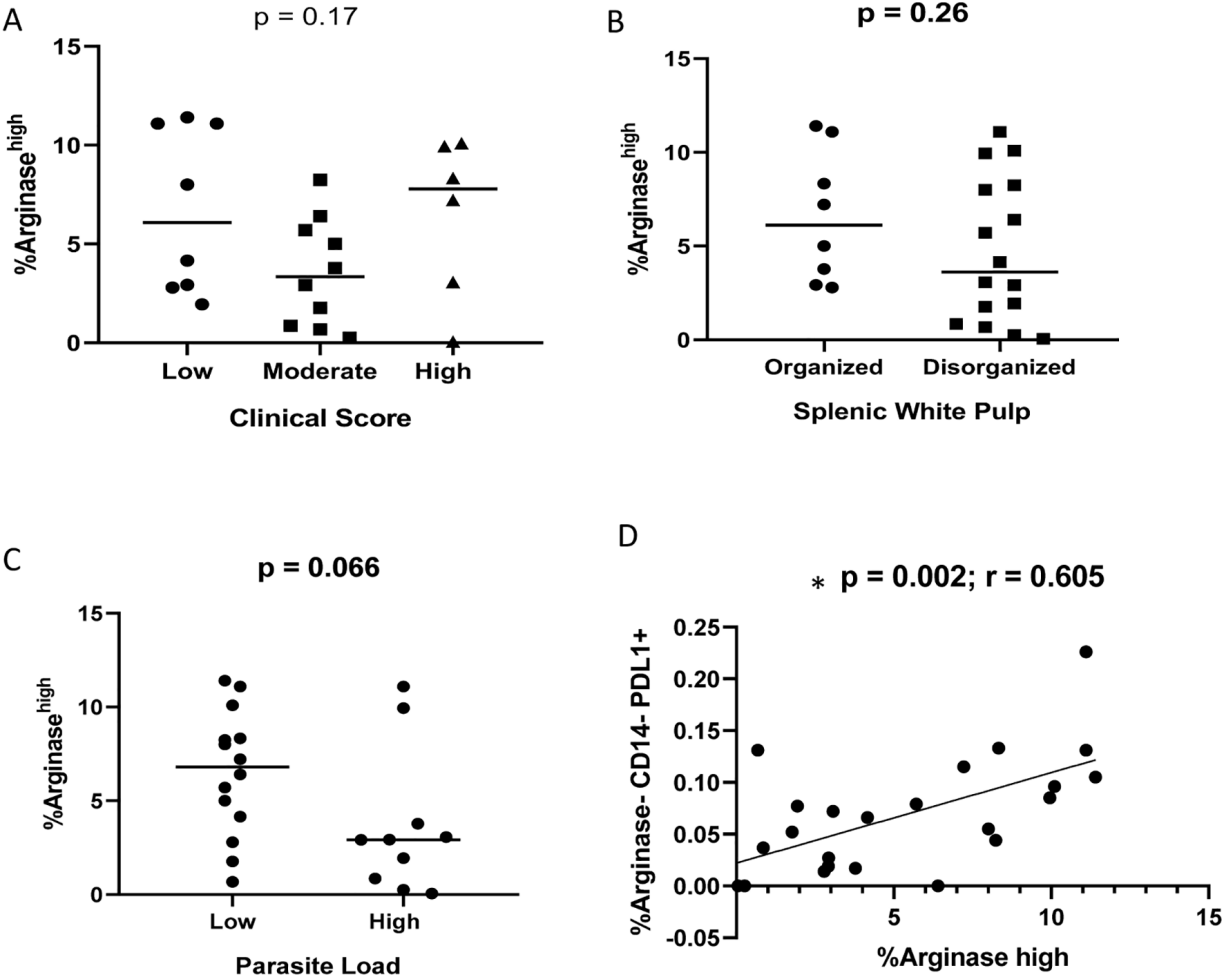
Percentage of Arginase-1^high^ cells according to **(A)** clinical score (Kruskal-Wallis test); **(B)** splenic white pulp disorganization (Unpaired t test); **(C)** parasite load (Mann-Whitney test). **(D)** correlation between CD14^-^arginase-1^-^PD-L1^+^ and arginase-1^high^ cells (Spearman correlation).

Flow cytometric analysis revealed the presence of arginase-1^high^ and arginase-1^+^PD-L1^+^ co-expressing cells in the spleens of naturally infected dogs (**Supplementary Table S3**). Although some of them were CD14^+^ cells, it was not possible to define the other arginase-1^high^ cells due to the absence of additional profile markers in our experiments. No statistically significant differences were observed according to clinical score, parasite load and splenic white pulp organization groups (**Figure 7**). However, a correlation between CD14^-^arginase-1^-^PD-L1^+^ and arginase-1^high^ cells was observed (p = 0.002; r = 0.605, Spearman correlation) (**Figure 7D**).

## Discussion

This study presents a comprehensive immunopathological evaluation of macrophage focused regulation and pro-exhaustion profiles in the spleens of 34 dogs naturally infected with *Leishmania infantum*. Through integrated analysis of clinical data, splenic white pulp disorganization (SWPD), and parasite load quantification, we demonstrate concurrent activation of both M1 (NOS2^+^) and M2 (TGF-β^+^, CD206^+^, Arginase-1^+^, pSTAT3^+^) macrophage phenotypes in infected spleens. Notably, elevated CD206 expression correlated with SWPD severity and parasite load, suggesting a pivotal role for M2 polarization in canine visceral leishmaniasis (CVL) immunopathogenesis. A lower CD68^+^NOS2^+^/CD68^+^arginase-1^+^ cells ratio was observed in spleens from dogs with high parasite load, indicating a potential role of M1/M2 balance in the failure to control *Leishmania* replication. Since NOS2 is directly associated with nitric oxide (NO) production—a key effector molecule for macrophage-mediated parasite killing—while arginase-1 promotes polyamine production that supports parasite replication (35), a reduction in this ratio may favor *Leishmania* survival, even in the context of intense inflammation within the spleen. In this context, arginase-1 expression by macrophages was consistently high across all groups. Amastigotes were observed infecting both arginase-1-positive and -negative cells. Arginase-1, by converting arginine into ornithine and polyamines, creates a favorable microenvironment for the replication of *Leishmania* spp (32). Thus, the presence of arginase-1^+^ cells amid the intense inflammatory infiltrate suggests that these macrophages may serve as protected replication niches for the parasite. This could promote persistent infection and continuous immune stimulation, contributing to chronic tissue damage characterized by splenic white pulp disorganization. Our data suggest a strong Th2/M2 immune profile in the spleens of dogs with VL caused by *L. infantum*, which may promote parasite replication and reflect an immunosuppressive microenvironment. Herein, we evidenced a positive correlation between CD206^+^ cells and amastigotes. Notably, both CD68^+^PD-L1^+^ and CD206^+^ cells were more abundant in animals with disorganized splenic white pulp. This aligns with findings in a murine model of cysticercosis, a helminth infection, where early M2 differentiation was capable of suppressing T-cell responses via PD-L1 and PD-L2 through direct cell-cell interaction (36). The authors suggested that the stimulation of M2 differentiation deactivates the damaging inflammatory response (36), and maybe parasites like *Taenia* sp or *Leishmania* sp might exploit such regulatory mechanisms to escape immune clearance and ensure persistence. It has been hypothesized that during chronic *Leishmania* infection, excessive arginase-1 release by macrophages could contribute to T lymphocytes exhaustion (35).

In our study, we observed for the first time a high arginase-1-expressing cell population positively correlated with PD-L1 expression, indicating a possible synergistic role between the arginase-1^high^ expressing cells and exhaustion pathways. Some of these were identified as CD14^+^, however other CD14^-^ cells could also express arginase-1^high^. The data suggest that not only macrophages, but others arginase-1^high^ expressing cell types may contribute for suppressive/regulatory profile during immunopathogenesis of canine visceral leishmaniasis. Future studies are needed to really answer this hypothesis and to identify which cells is expressing arginase-1^high^ since we were not able to characterize this population. Interestingly, previous studies in HIV-1-infected patients and inflammatory myopathies have reported PD-L1^+^/arginase-1^+^ neutrophils (37, 38). In inflammatory myopathies, myeloid-derived suppressor cells (MDSCs)—comprising monocytes and polymorphonuclear cells—co-expressing PD-L1 and arginase-1 were associated with disease progression (37). The authors demonstrated the PD-L1 and arginase-1 co-expression, as well as arginase-1^high^ expression by MDSCs (39). Maybe these cells could also play a role in immunopathogenesis of visceral leishmaniasis since inflammatory persistent stimulus can generate these immature myeloid cells with regulatory functions. Further investigation is needed to test this hypothesis.

In the present study we also demonstrated pSTAT3 expression in CD68^+^ cells in the spleen of dogs with CVL, with a significant correlation with the arginase-1 pathway. Studies showed a direct correlation between pSTAT3-mediated regulation of PD-L1 expression in tumor cells *in vitro* (40). In addition, the silencing of this protein negatively regulated PD-L1 expression in tumor cells and resulted in a direction towards an M1 phenotype of tumor-associated macrophages marked by elevated MHC II expression (40). In our study, the correlation between arginase-1 expression and PD-L1^+^ cells corroborating the potential interaction by M2 and exhaustion pathways, and the possible impact of them in immunopathogenesis and parasite survival. These pathways may be activated due to various factors associated with the inflammatory process generated by *L. infantum* infection or directly by the parasite itself (38).

We hypothesized that even in small quantities, a PD-L1^+^ cell can engage PD-1 receptors in multiple lymphocytes, triggering the process of cellular exhaustion. According to the literature, *in vitro* and *in vivo* blockade of PD-L1 resulted in decreased parasite survival and increased production of IL-12, TNF, ROS, and NO by macrophages (39) indicating that *Leishmania* parasites take advantage of the PD-1 pathway not only for T cell exhaustion, but also to prevent macrophage killing mechanisms and death, thus favoring parasite persistence. The authors showed the blockade of PD-L1 reduces arginase-1 in macrophages and polyamine availability to parasites, and favors proinflammatory macrophage activation and *Leishmania* control. Herein, a tendency to increased expression of PD-L1^+^ macrophages was observed in dogs with disorganized spleen microarchitecture, suggesting a role of exhaustion pathway in the immunopathogenesis of CVL and parasite persistence.

As future trends, we seek to identify profile and functional markers for M1 and M2 macrophages that would allow the evaluation of parasite load in both cell types. In this sense, quantifying the number of infected cells and the number of amastigotes per macrophage with distinct profiles could contribute to the understanding of spleen infection in naturally infected dogs. Similarly, we aim to identify specific markers for dogs to assess the role of immature myeloid cells in this process. These cells express high levels of arginase-1 and PD-L1 and are involved in the pathogenesis of various inflammatory diseases (37, 38). Their role in visceral leishmaniasis has not yet been verified. We believe that the arginase-1 and cellular exhaustion pathways stimulate each other and constitute interesting targets for immunomodulation in visceral leishmaniasis.

## Conclusions

There is a fine balance between M1 and M2 cells, and as the disease progresses, these populations become quantitatively closer. However, When the parasitic load is lower, there is a predominance of M1 cells. The persistence and expansion of cell populations displaying an M2-like phenotype (e.g., CD206^+^, arginase-1^+^, pSTAT3^+^, TGF-β^+^) within the intense inflammatory infiltrate of spleens parasitized by *Leishmania* may be a key factor in parasite persistence and the chronic stimulation of the immune response in naturally infected dogs. This ongoing immune activation likely contributes to chronic tissue damage, marked by splenic white pulp disorganization and exacerbation of clinical signs associated with visceral leishmaniasis.

Furthermore, the involvement of co-expressing Arginase-1^+^PD-L1^+^ cells was observed suggesting an association between M2 profile and cell exhaustion showing a cooperation by both pathways in CVL. The results of this study suggest that the predominant microbicidal response of M1 macrophages in relation to regulation by M2 macrophages, together with the blockade of cell exhaustion pathways, may be crucial for the effective control of visceral leishmaniasis. These insights could contribute to the development of more effective treatment strategies against this potentially fatal disease.

## Data Availability

The original contributions presented in the study are included in the article/**Supplementary Material**. Further inquiries can be directed to the corresponding authors.

## Supplementary material

Supplementary Table 1 Target genes and their sequences used in quantification of the parasite load. S, sense; AS, antisense, bp, base pairs.

Supplementary Table 2 Quantitative analysis of functional markers of M1, M2 and PD-L1^+^ macrophages according to clinical score, parasite load and splenic white pulp disorganization of dogs naturally infected with *L. infantum*. Data are represented as median (minimum-maximum values).

Supplementary Table 3 Analysis of immunological markers in spleen of dogs naturally infected with *Leishmania infantum* according to clinical score, parasite load and splenic white pulp disorganization using flow cytometer technique. Data are represented by median and minimum and maximum values.

Supplementary Figure 1 Gate strategy. **(A)** Forward scatter area (FSC-A) *vs*. side scatter (SSC-A) dot plot with population gate (Cels.); **(B)** Forward scatter area (FSC-A) *vs*. forward scatter height (FSC-H) dot plot from gate Cels with Singlets gate; **(C)** Histogram of Zombie-KO525 staining within Singlets gate showing living cells (Alive). **(D)** Histogram of CD14-APC within Alive gate staining showing CD14^+^ cells; **(E)** Histogram of Arginase-PECY7 staining within Alive gate showing Arginase-1^+^ cells and Arginase ^high^; **(F)** Histogram of PD-L1-PE staining within Alive gate showing PD-L1^+^ cells within Alive gate.
