# Supplementary material for: Splenic macrophage functional profile and its role in the immunopathogenesis of canine visceral leishmaniasis
Source: Front Immunol. 2025 Jun 20;16:1617751. doi: 10.3389/fimmu.2025.1617751 (PMC12226308; doi:10.3389/fimmu.2025.1617751)
Supplement: Supplementary Table 1 — Target genes and their sequences used in quantification of the parasite load. S, sense; AS, antisense, bp, base pairs. [file Table1.docx]

**Supplementary Table 1.** HPRT and SSU rRNA gene sequences used for quantification of the parasite load.

| Target Gene | Sequence (5'-3') | Base pair size (bp) |
| --- | --- | --- |
| HPRT Canine target (NW_003726126.1) | AAAACAATGCAGACTTTGCT  CCTTGACCATCTTTGGATTA | 58 |
| SSU rRNA Parasite target Leishmania (L). infantum  (Prina et al. (2007)) | TACTGGGGCGTCAGAG  GGGTGTCATCGTTTGC | 153 |

bp, base pairs
