# Supplementary material for: Splenic macrophage functional profile and its role in the immunopathogenesis of canine visceral leishmaniasis
Source: Front Immunol. 2025 Jun 20;16:1617751. doi: 10.3389/fimmu.2025.1617751 (PMC12226308; doi:10.3389/fimmu.2025.1617751)
Supplement: Supplementary Table 2 — Quantitative analysis of functional markers of M1, M2 and PD-L1+ macrophages according to clinical score, parasite load and splenic white pulp disorganization of dogs naturally infected with L. infantum. Data are represented as median (minimum-maximum values). [file Table2.docx]

**Supplementary Table 2**

Supplementary Table 2. Quantitative analysis of functional markers of M1, M2 and PD-L1^+^ macrophages according to clinical score, parasite load and splenic white pulp disorganization of dogs naturally infected with *L. infantum*. Data are represented as median (minimum-maximum values)

| Clinical Score | | | | |
| --- | --- | --- | --- | --- |
| Marker | Low | Medium | | High |
| NOS2%* | 40.94 (8.26 – 47.96) | 40.81 (3.08 – 57.56) | | 28.7 (8.23 – 54.17) |
| NOS2 mm^2*^ | 1,116.0 (231.1 – 1,680.0) | 1,202.0 (85.33 – 2.308.0) | | 848.9 (193.8 – 1,694.0) |
| Mannose receptor%* | 29.91 (10.62 – 37.25) | 36.26 (20.99 – 49.43) | | 30.48 (13.04 – 50.81) |
| Mannose receptor mm^2*^ | 510.2 (174.2 – 696.9) | 609.8 (256.0 – 1,228.0) | | 512.0 (147.6 – 776.9) |
| TGF-β%* | 28.32 (12.85 – 45.73) | 27.51 (14.83 – 37.28) | | 27.14 (9.15 – 33.15) |
| TGF-β mm^2*^ | 497.8 (165.3 – 837.3) | 456.9 (280.0 – 629.3) | | 472.9 (81.78 – 750.2) |
| Macrophage^+^ STAT-3P^+^%** | 84.96 (29.14 – 96.51) | 84.76 (53.29 – 93.62) | | 68.13 (29.54 – 94.78) |
| Macrophage^+^ arginase 1^+^%** | 83.51 (65.16 – 93.72) | 70.27 (37.52 – 100.0) | | 83.33 (34.78 – 100.0) |
| Macrophage^+^ NOS2^+^%** | 98.55 (86.52 – 100.0) | 95.57 (61.29 – 100.0) | | 97.40 (75.0 – 100.0) |
| CD68^+^ PDL1^+^%** | 0.42 (0 – 5.99) | 1.49 (0.7 – 5.49) | | 0.33 (0 – 15.08) |
| CD68^+^ PDL1^+^mm^2**^ | 17.78 (0 – 257.8) | 124.4 (35.56 – 284.4) | | 8.89 (0 – 844.4) |
| Amastigotes mm^2*^ | 205.6 (113.3 – 494.4) | 191.1 (0 – 1,022.0) | | 146.7 (31.11 – 808.9) |
| Parasite load | | | | |
| Marker | Low parasite load | | High parasite load | |
| NOS2%* | 39.94 (8.23 – 54.17) | | 40.85 (3.08 – 57.56) | |
| NOS2 mm^2*^ | 1,000.0 (193.8 – 1,680.0) | | 1276.0 (85.33 – 2,308.0) | |
| Mannose receptor%* | 31.37 (10.62 – 50.81) | | 30.84 (18.8 – 43.27) | |
| Mannose receptor mm^2*^ | 563.6 (147.6 – 776.9) | | 522.7 (256.0 – 1,228.0) | |
| TGF-β%* | 25.79 (9.15 – 45.73) | | 28.46 (12.85 – 36.34) | |
| TGF-β mm^2*^ | 439.1 (81.78 – 837.3) | | 518.2 (165.3 – 750.2) | |
| Macrophage^+^ STAT-3P^+^%** | 79.17 (29.14 – 95.79) | | 72.96 (29.54 – 96.51) | |
| Macrophage^+^ arginase 1^+^%** | 72.96 (34.78 – 100.0) | | 86.24 (43.2 – 100.0) | |
| Macrophage^+^ NOS2^+^%** | 98.21 (86.52 – 100.0) | | 95.57 (61.29 – 100.0) | |
| CD68^+^ PDL1^+^%** | 0.87 (0 – 5.99) | | 1.28 (0 – 15.08) | |
| CD68^+^ PDL1^+^mm^2**^ | 44.44 (0 – 284.4) | | 62.22 (0 – 844.4) | |
| Splenic White Pulp Organization | | | | |
| Marker | Organized to slight disorganization | | Moderate to intense disorganization | |
| NOS2%* | 33.36 (10.77 – 54.17) | | 40.34 (3.08 – 57.56) | |
| NOS2 mm^2*^ | 1,120.0 (247.1 – 1,515.0) | | 1,230.0 (85.33 – 2,308.0) | |
| Mannose receptor%* | 28.99 (13.04 – 40.28) | | 36.21 (10.62 – 50.81) | |
| Mannose receptor mm^2*^ | 512.0 (147.6 – 696.9) | | 567.1 (174.2 – 1,228.0) | |
| TGF-β%* | 25.41 (9.15 – 33.06) | | 28.27 (14.83 – 45.73) | |
| TGF-β mm^2*^ | 414.2 (81.78 – 718.2) | | 512.0 (327.1 – 837.3) | |
| Macrophage^+^ STAT-3P^+^%** | 78.51 (37.44 – 96.51) | | 72.96 (29.14 – 94.39) | |
| Macrophage^+^ arginase 1^+^%** | 82.84 (34.78 – 100.0) | | 70.45 (37.52 – 100.0) | |
| Macrophage^+^ NOS2^+^%** | 97.74 (90.88 – 100.0) | | 96.86 (61.29 – 100.0) | |
| CD68^+^ PDL1^+^%** | 0.38 (0 – 3.19) | | 1.28 (0 – 15.08) | |
| CD68^+^ PDL1^+^mm^2**^ | 17.78 (0 – 124.4) | | 257.8 (0 – 844.4) | |
| Amastigotes mm^2*^ | 146,7 (31.11 – 408.9) | | 218.9 (0 – 1,022.0) | |

* Data obtained using immunohistochemistry

** Data obtained using immunofluorescence
