# Supplementary material for: Splenic macrophage functional profile and its role in the immunopathogenesis of canine visceral leishmaniasis
Source: Front Immunol. 2025 Jun 20;16:1617751. doi: 10.3389/fimmu.2025.1617751 (PMC12226308; doi:10.3389/fimmu.2025.1617751)
Supplement: Supplementary Table 3 — Analysis of immunological markers in spleen of dogs naturally infected with Leishmania infantum according to clinical score, parasite load and splenic white pulp disorganization using flow cytometer technique. Data are represented by median and minimum and maximum values. [file Table3.docx]

**Supplementary Table 3**

Supplementary Table 3. Analysis of immunological markers in spleen of dogs naturally infected with *Leishmania infantum* according to clinical score, parasite load and splenic white pulp disorganization using flow cytometer technique. Data are represented by median and minimum and maximum values.

| Marker (%) | Low clinical score | Medium clinical score | | High clinical score |
| --- | --- | --- | --- | --- |
| Arginase | 73.7 (69.1 – 79.4) | 73.35 (65.5 – 78.5) | | 69.9 (67.0 – 75.4) |
| Arginase high | 6.08 (1.94 – 11.4) | 3.35 (0.26 – 8.24) | | 7.78 (0.056 – 10.10) |
| CD14 | 2.38 (0.67 – 3.8) | 1.09 (0.28 – 4.76) | | 0.44 (0.12 – 1.41) |
| PD-L1 | 0.29 (0.12 – 1.27) | 0.14 (0.054 – 0.64) | | 0.32 (0.19 – 0.56) |
| Arginase+ CD14+ PDL1+ | 0.016 (0.005 – 0.12) | 0.012 (0.002 – 0.051) | | 0.041 (0.003 – 0.067) |
| Arginase+ CD14+ PDL1- | 1.94 (0.072 – 3.42) | 0.77 (0.26 – 3.36) | | 0.32 (0.10 – 1.04) |
| Arginase+ CD14- PDL1+ | 0.083 (0.07 – 0.95) | 0.067 (0.037 – 0.48) | | 0.18 (0.097 – 0.45) |
| Arginase+ CD14- PDL1- | 72.80 (68.5 – 77.6) | 70.8 (65.1 – 74.6) | | 69.3 (65.8 – 74.9) |
| Arginase- CD14+ PDL1+ | 0.011 (0 – 0.021) | 0.003 (0 – 0.04) | | 0.002 (0 – 0.03) |
| Arginase- CD14+ PDL1- | 0.32 (0.014 – 0.81) | 0.17 (0.004 – 0.48) | | 0.076 (0.005 – 0.27) |
| Arginase- CD14- PDL1+ | 0.072 (0.014 – 0.23) | 0.037 (0 – 0.13) | | 0.091 (0 – 0.13) |
| Arginase- CD14- PDL1- | 24.35 (0 – 30.7) | 27.8 (0 – 34.3) | | 29.95 (24.5 – 32.6) |
| Marker (%) | Low parasite load | | High parasite load | |
| Arginase | 72.5 (65.5 – 79.4) | | 73.55 (69.1 – 78.5) | |
| Arginase high | 6.81 (0.68 – 11.4) | | 2.93 (0.056 – 11.10) | |
| CD14 | 1.48 (0.12 – 3.99) | | 1.045 (0.25 – 4.76) | |
| PD-L1 | 0.23 (0.054 – 1.27) | | 0.25 (0.063 – 0.58) | |
| Arginase+ CD14+ PDL1+ | 0.012 (0.002 – 0.12) | | 0.024 (0.003 – 0.096) | |
| Arginase+ CD14+ PDL1- | 0.94 (0.072 – 3.42) | | 0.69 (0.19-3.36) | |
| Arginase+ CD14- PDL1+ | 0.11 (0.05 – 0.95) | | 0.12 (0.037 – 0.48) | |
| Arginase+ CD14- PDL1- | 70.70 (65.1 – 77.6) | | 70.65 (66.3 – 75.0) | |
| Arginase- CD14+ PDL1+ | 0.009 (0 – 0.03) | | 0.0015 (0 – 0.04) | |
| Arginase- CD14+ PDL1- | 0.17 (0.005 – 0.81) | | 0.15 (0.004 – 0.38) | |
| Arginase- CD14- PDL1+ | 0.079 (0 – 0.23) | | 0.032 (0 – 0.13) | |
| Arginase- CD14- PDL1- | 27.8 (20.1 – 34.3) | | 26.15 (0 – 33.2) | |
| Marker (%) | Organized splenic White pulp ^#^ | | Disorganized splenic White pulp ^#^ | |
| Arginase | 71.5 (67.0 – 75.4) | | 73.7 (65.5 – 79.4) | |
| Arginase high | 6.12 (2.79 – 11.4) | | 3.62 (0.056 – 11.10) | |
| CD14 | 1.48 (0.48 – 3.99) | | 1.09 (0.12 – 4.76) | |
| PD-L1 | 0.31 (0.063 – 0.81) | | 0.19 (0.054 – 1.27) | |
| Arginase+ CD14+ PDL1+ | 0.04 (0.007 – 0.12) | | 0.015 (0.002 – 0.075) | |
| Arginase+ CD14+ PDL1- | 0.85 (0.072 – 1.82) | | 0.69 (0.1 – 3.42) | |
| Arginase+ CD14- PDL1+ | 0.11 (0.037 – 0.18) | | 0.10 (0.042 – 0.95) | |
| Arginase+ CD14- PDL1- | 68.9 (65.8 – 77.6) | | 71.2 (65.1 – 75.0) | |
| Arginase- CD14+ PDL1+ | 0.011 (0 – 0.03) | | 0.0025 (0 – 0.04) | |
| Arginase- CD14+ PDL1- | 0.17 (0.014 – 0.36) | | 0.19 (0.004 – 0.81) | |
| Arginase- CD14- PDL1+ | 0.11 (0.014 – 0.13) | | 0.061 (0 – 0.23) | |
| Arginase- CD14- PDL1- | 29.2 (22.2 – 32.6) | | 25.85 (0 – 34.3) | |

# Organized splenic White pulp: organized to slightly disorganized; Disorganized splenic White pulp: moderate to intense disorganization
