# Supplementary figures and images for: Splenic macrophage functional profile and its role in the immunopathogenesis of canine visceral leishmaniasis
Source: Front Immunol. 2025 Jun 20;16:1617751. doi: 10.3389/fimmu.2025.1617751 (PMC12226308; doi:10.3389/fimmu.2025.1617751)

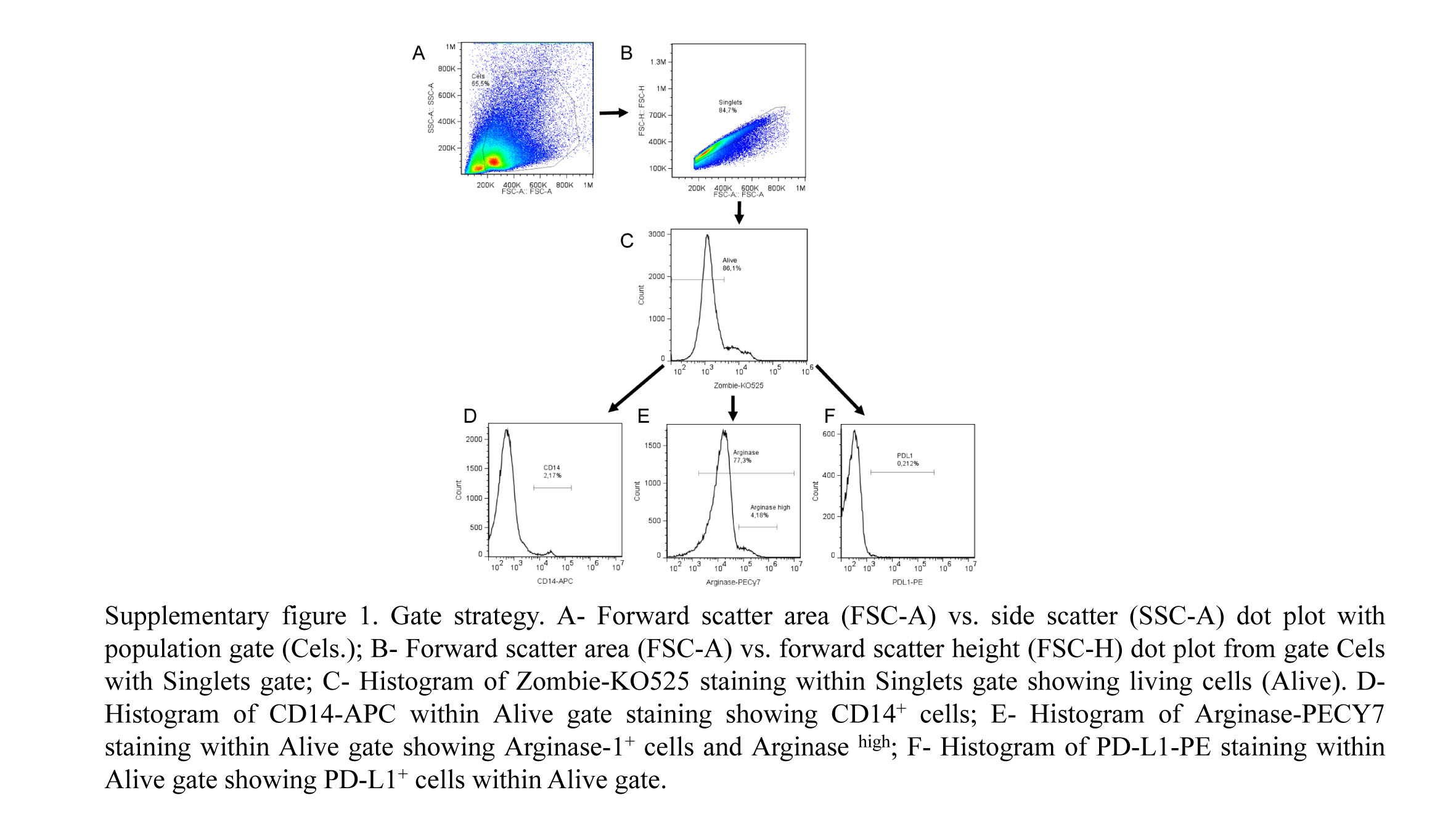

Supplement: Supplementary Figure 1 — Gate strategy. (A) Forward scatter area (FSC-A) vs. side scatter (SSC-A) dot plot with population gate (Cels.); (B) Forward scatter area (FSC-A) vs. forward scatter height (FSC-H) dot plot from gate Cels with Singlets gate; (C) Histogram of Zombie-KO525 staining within Singlets gate showing living cells (Alive). (D) Histogram of CD14-APC within Alive gate staining showing CD14+ cells; (E) Histogram of Arginase-PECY7 staining within Alive gate showing Arginase-1+ cells and Arginase high; (F) Histogram of PD-L1-PE staining within Alive gate showing PD-L1+ cells within Alive gate. [file Image1.jpg]
